# Modelling the effects of chloroquine on *KCNJ2*-linked short QT syndrome

**DOI:** 10.18632/oncotarget.22490

**Published:** 2017-11-18

**Authors:** Cunjin Luo, Kuanquan Wang, Henggui Zhang

**Affiliations:** ^1^ School of Computer Science and Technology, Harbin Institute of Technology (HIT), Harbin, China; ^2^ School of Physics and Astronomy, The University of Manchester, Manchester, United Kingdom; ^3^ Space Institute of Southern China, Shenzhen, China; ^4^ Key Laboratory of Medical Electrophysiology, Ministry of Education, Collaborative Innovation Center for Prevention and Treatment of Cardiovascular Disease/Institute of Cardiovascular Research, Southwest Medical University, Luzhou, China

**Keywords:** arrhythmia, short QT syndrome (SQTS), inward rectifier, chloroquine (CQ), computer modelling

## Abstract

A gain-of-function *KCNJ2* D172N mutation in KCNJ2-encoded Kir2.1 channels underlies one form of short QT syndrome (SQT3), which is associated with increased susceptibility to arrhythmias and sudden death. Anti-malarial drug chloroquine was reported as an effective inhibitor of Kir2.1 channels. Using biophysically-detailed human ventricle computer models, this study assessed the effects of chloroquine on SQT3. The ten Tusscher *et al.* model of human ventricular cell action potential was modified to recapitulate functional changes in the inward rectifier K^+^ current (*I*_K1_) due to heterozygous and homozygous forms of the D172N mutation. Mutant formulations were incorporated into multi-scale models. The blocking effects of chloroquine on ionic currents were modelled using IC_50_ and Hill coefficient values from literatures. Effects of chloroquine on action potential duration (APD), effective refractory period (ERP) and pseudo-ECGs were quantified. It was shown that chloroquine caused a dose-dependent reduction in *I*_K1_, prolonged APD, and decreased the maximum voltage heterogeneity. Chloroquine prolonged QT interval and declined the T-wave amplitude. Although chloroquine reduced tissue’s temporal vulnerability, it increased the minimum substrate size necessary for sustaining re-entry. The actions of chloroquine decreased arrhythmia risk, due to the reduced tissue vulnerability, prolonged ERP and wavelength of re-entrant excitation waves, which in combination prevented and terminated re-entry in the tissue models. In conclusion, the results of this study provide new evidence that the anti-arrhythmic effects of chloroquine on SQT3 and, by extension, to the possibility that chloroquine may be a potential therapeutic agent for SQT3 treatment.

## INTRODUCTION

Short QT syndrome (SQTS) is an inherited disorder referring to the electrocardiographic manifestation of accelerated cardiac repolarization. It was first reported as a distinct clinical entity, and was suggested an association with atrial and ventricular fibrillation in 2000 [1]. The familial nature and arrhythmogenic potential of SQTS were confirmed by Gaita *et al.* in 2003 [2]. SQTS is a rare and autosomal dominant disease that manifests with arrhythmias, sudden cardiac death (SCD) and abnormally shortened QT intervals on the electrocardiogram (ECG) [1–4]. To date, six different genes encoding various cardiac ion channels have been identified in the pathogenesis of SQTS, including the *KCNH2* [5], *KCNQ1* [6], *KCNJ2* [7], *CACNA1C* [8], *CACNB2B* [8] and *CACNA2D1* genes [9]. Data regarding the genotype-phenotype relationship and its pharmacological treatment are promising but limited, primarily due to the lack of clinical cases and experimental models.

The SQTS variant 3 (SQT3) is caused by a mutation in *KCNJ2* gene encoding the subunit of Kir2.1 of the inward rectifier K^+^ current (*I*_K1_). Priori and colleagues [7] described a missense mutation (D172N) in a juvenile proband and her father with abbreviated QT intervals (315 ms and 320 ms, respectively) and an abnormal T-wave morphology, and with a history of pre-syncopal events and palpitations. Functional analysis revealed that D172N mutation led to changes in the voltage-dependent properties of *I*_K1_, which results in a gain-of-function on the Kir2.1 channel, producing a significant increase of *I*_K1_ [7]. For the D172N *KCNJ2* SQT3 mutation, the previous study was able to reproduce the shortening of the action potential (AP) and initiation and maintenance of re-entrant excitation waves in computational models of human ventricular electrophysiology [10].

Given the relatively high risk of SCD from fatal arrhythmic events and a high penetrance in affected families (∼31%) [11], an implantable cardioverter defibrillator (ICD) is considered as the primary therapy for these patients [12, 13]. However, ICD therapy is expensive, and ICD placement is not always practical or technically feasible. To efficiently treat SQTS patients, genotype-specific pharmacological treatment in SQTS would be beneficial. Anti-malarial drug chloroquine (CQ) was reported as an effective inhibitor of Kir2.1 channels, and it may be a potential therapeutic agent for SQT3 treatment [14]. CQ was identified as an effective pharmacological inhibitor of both D172N [14] and WT-D172N [15] mutant Kir2.1 channels. However, it is uncertain whether or not results obtained at a single-cell level can be extrapolated to the propagation of the excitation wave in cardiac tissue. Furthermore, it did not consider the potential effect of the combination of rapid delayed rectifier K^+^ current (*I*_Kr_) and *I*_K1_ blockade, which was found in experiments with the use of CQ [16].

Due to a lack of phenotypically accurate experimental models, there has hitherto not been any detailed investigation of how the antiarrhythmic drug influences ventricular cellular and tissue electrophysiology in SQTS. In recent years, there has been a tremendous research effort in the development of biophysically detailed computer models of the heart [17–21]. These models have been extensively implemented as a cardiac platform for investigating the functions of the heart during various physiological, pathological and pharmacological conditions [22–25]. Moreover, these models have also been employed successfully in our previous studies to dissect ionic mechanisms underlying QT interval shortening and pro-arrhythmia in SQTS [10, 26–28]. Accordingly, this study aimed to assess the potential effects of CQ on ventricular electrical excitation associated with SQT3, using heterogeneous, multi-scale models of human ventricular electrophysiology [29].

## RESULTS

### Effects of chloroquine on SQT3 in single cells

**Figure 1:**
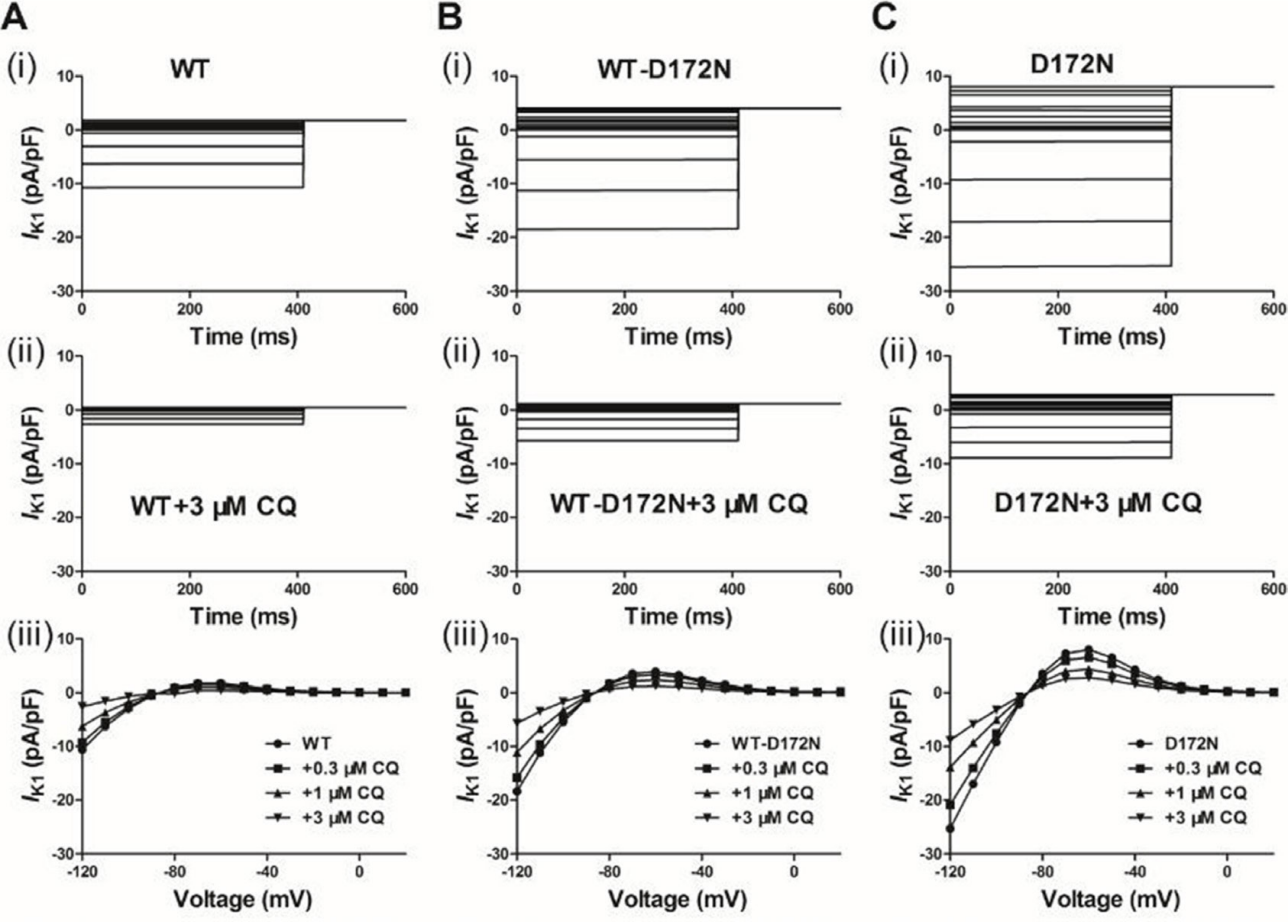
Simulated action potential clamp experiments CQ blocks *I*_K1_ current through the WT and mutant WT-D172N and D172N Kir2.1 channels. (**A**) *I*_K1_ current traces for the WT (i) and WT + 3 µM CQ (ii) conditions elicited by a 400 ms pulses from –120 to 20 mV, applied in 10 increments, and I-V relationships for the peak current in the WT condition and the presence of CQ at 0.3, 1 and 3 µM (iii). (**B**) *I*_K1_ current traces for the WT-D172N (i) and WT-D172N + 3 µM CQ (ii) conditions and I-V relationships for the peak current in the WT-D172N condition and the presence of CQ at 0.3, 1 and 3 µM (iii). (**C**) *I*_K1_ current traces for the D172N (i) and D172N + 3 µM CQ (ii) conditions and I-V relationships for the peak current in the WT condition and the presence of CQ at 0.3, 1 and 3 µM (iii).

First, we tested the ability of the *I*_K1_ model to reproduce the previously published experimental effects of CQ on the WT and mutant Kir2.1 *I*_K1_ at physiological temperature [14, 16]. Figure 1 shows the voltage clamp protocol used which is the same as that used for the experimental data [14, 16] and the generated *I*_K1_ current traces with the actions of CQ from which the I-V were reconstructed. The simulated I-V relationships for the WT and mutant WT-D172N and D172N conditions match those recorded experimentally [14, 16]. The cell model was elicited by 400 ms depolarizing voltage steps from –120 to +20 mV and from a holding potential of –60 mV, with current traces for the WT simulations at the indicated CQ concentrations (0.3, 1, and 3 µM), as shown in Figure 1A(i-iii). For the WT condition, small outward but large inward *I*_K1_ was evoked. Figure 1B(i-iii) and 1C(i-iii) shows I-V data for the WT-D172N and D172N simulations at the indicated CQ concentrations, respectively. D172N Kir2.1 outward current reached a higher peak amplitude, and WT-D172N Kir2.1 current was intermediate. Similar to WT Kir2.1, CQ reduced WT-D172N and D172N Kir2.1 current in a voltage-dependent manner.

**Figure 2:**
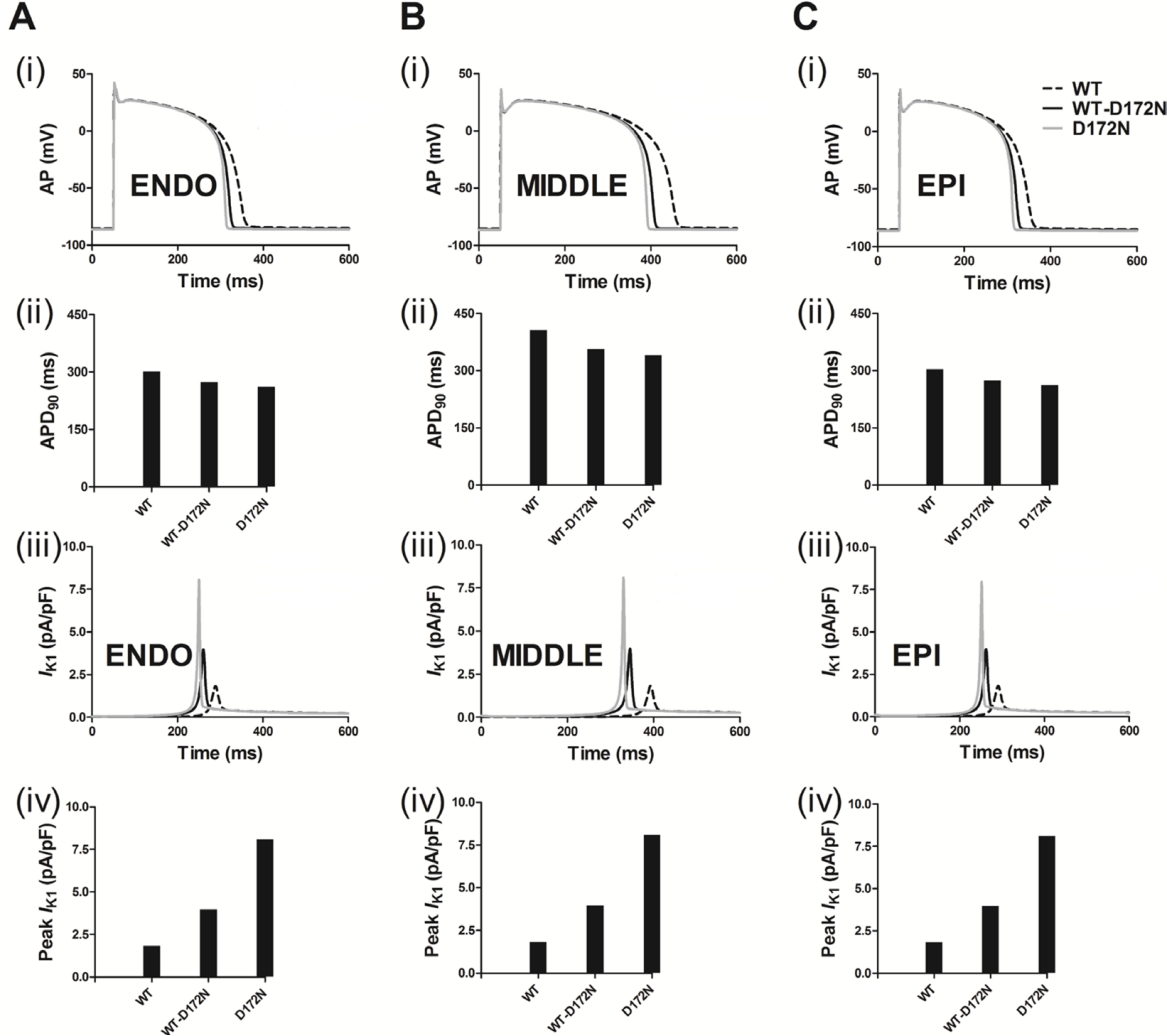
Simulated transmembrane voltages of ventricular myocyte together with the corresponding *I*_K1_ current profile and a peak density for the WT, WT-D172N, and D172N conditions Gain-of-function of ***I***_**K1**_ abbreviated the AP. (**A**) ENDO simulations of AP waveforms (i), corresponding APD_90_ histogram (ii), *I*_K1_ current profile (iii) and peak density (iv) in the WT, WT-D172N, and D172N conditions. (**B**) MIDDLE simulations of AP waveforms (i), corresponding APD_90_ histogram (ii), *I*_K1_ current profile (iii), and peak density (iv) in the WT, WT-D172N, and D172N conditions. (**C**) EPI simulations of AP waveforms (i), corresponding APD_90_ histogram (ii), *I*_K1_ current profile (iii), and peak density (iv) in the WT, WT-D172N and D172N conditions.

Following incorporation of the WT and mutant WT-D172N and D172N formulations into the ten Tusscher *et al.* model [29], Figure 2A shows the simulated AP (i, ii) and *I*_K1_ profile (iii, iv) for an ENDO cell. The MIDDLE and EPI counterparts are shown in Figure 2B and 2C. From the results, the simulated *I*_K1_ in the Kir2.1 D172N homozygous condition dramatically shortened the human ventricular AP. Compared to the WT condition, the mutant model preserves characteristic spike and dome morphology of the human ventricular AP. Under the WT condition, the computed APD_90_ was 302, 406 and 304 ms for ENDO (Figure 2A), MIDDLE (Figure 2B) and EPI (Figure 2C) cells, respectively, which were shortened to 273, 357 and 274 ms for the heterozygous WT-D172N condition and to 261, 341 and 262 ms for the homozygous D172N condition. The D172N condition exerted a more profound effect than the WT-D172N condition. This was associated with a significantly increased outward *I*_K1_, providing a stronger repolarising current during the late phase of AP repolarization. The APD shortening in the mutant conditions was consistent with the greater conductance of mutant channels [14]. The observed resting potential (RP) values were –85.12, –84.80, and –85.10 mV for the ENDO, MIDDLE, and EPI cells, respectively, in the WT condition, which were slightly changed, respectively, to –85.84, –85.70, and –85.83 mV for the WT-D172N condition and to –86.18, –86.11, and –86.17 mV for the D172N condition.

**Figure 3:**
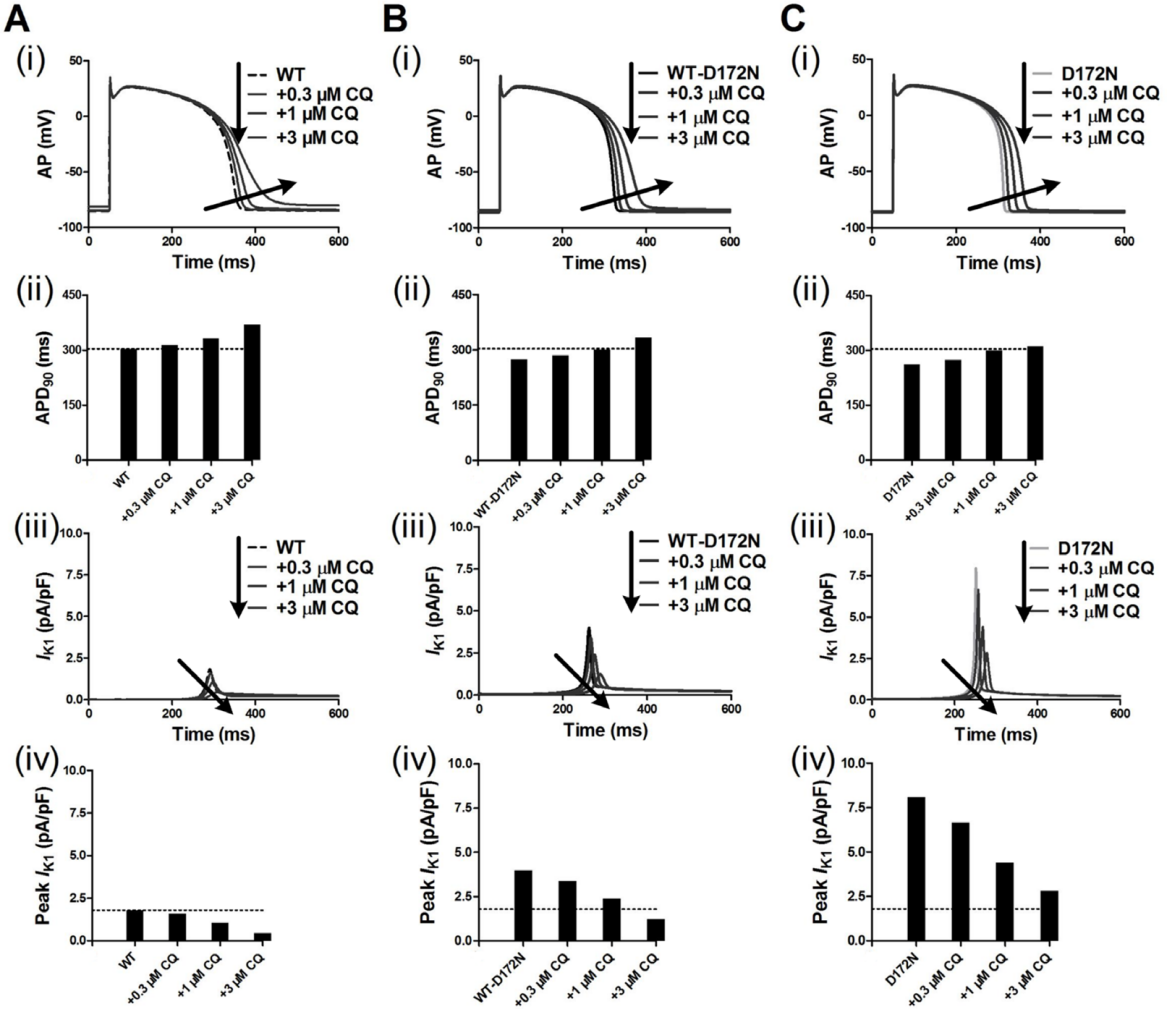
Transmembrane voltages of an EPI myocyte together with the corresponding *I*_K1_ current profile and peak density for the WT, WT-D172N, and D172N simulations at the indicated CQ concentrations (0.3, 1 and 3 µM) (**A**) AP waveforms (i), corresponding APD_90_ histogram (ii), *I*_K1_ current profile (iii), and peak density (iv) for the WT simulations at the indicated CQ concentrations. (**B**) AP waveforms (i), corresponding APD_90_ histogram (ii), *I*_K1_ current profile (iii), and peak density (iv) for the WT-D172N simulations at the indicated CQ concentrations. (**C**) AP waveforms (i), corresponding APD_90_ histogram (ii), *I*_K1_ current profile (iii), and peak density (iv) for the D172N simulations at the indicated CQ concentrations.

Figure 3 shows the effects of CQ on AP and *I*_K1_ during the AP time course for the Kir2.1 channels in an EPI ventricular myocyte. CQ caused a dose-dependent prolongation of APD_90_ and reduced the peak amplitude of *I*_K1_ for the WT and mutant WT-D172N and D172N conditions. The measured APD_90_ was prolonged from 274 ms under the WT-D172N condition to 286, 302, and 334 ms at the indicated CQ concentrations 0.3, 1, and 3 µM, respectively, and from 262 ms under the D172N condition to 274, 301, and 312 ms at the indicated CQ concentrations. The simulation results indicated that CQ concentration 3 µM was sufficient to normalize APD_90_ and peak *I*_K1_. Additional simulations on ventricular ENDO and MIDDLE cells (data not shown) showed similar effects of CQ to those seen with the ventricular EPI AP. The RP values were changed from –85.83 mV under the WT-D172N condition to –85.66, –85.20, and –83.74 mV at the indicated CQ concentrations 0.3, 1, and 3 µM, respectively, and from –86.17 mV under the D172N condition to –86.05, –85.71, and –85.14 mV at the indicated CQ concentrations.

**Figure 4:**
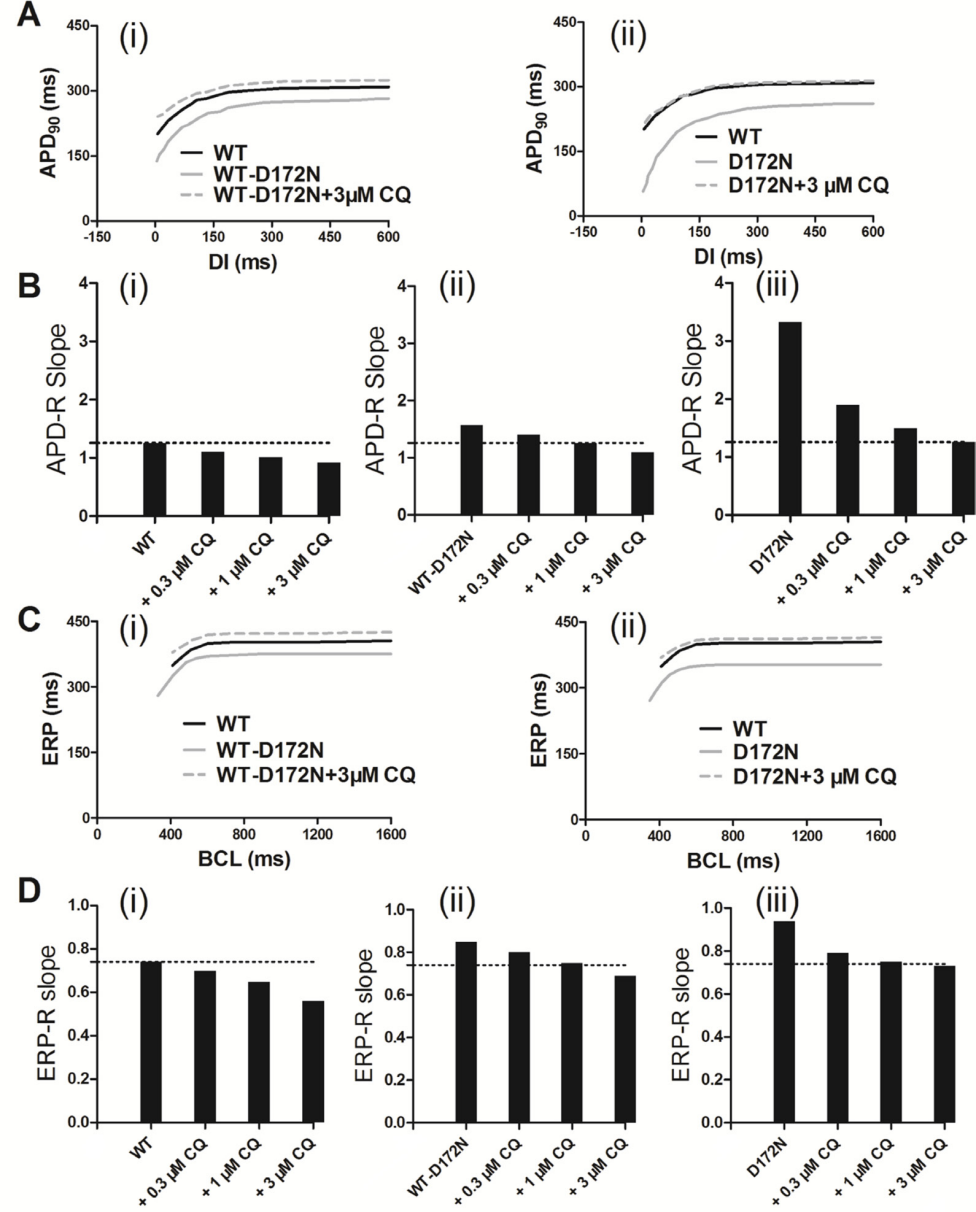
Rate-dependent APD restitution curves and ERP restitution curves for an EPI myocyte for the WT, WT-D172N, and D172N simulations at the indicated CQ concentrations (0.3, 1, and 3 µM) (**A**) APD restitution curves for the WT, WT-D172N (i), and D172N (ii) simulations at 3 µM concentration of CQ. (**B**) Measured slopes of APD restitution curves for the WT (i), WT-D172N (ii), and D172N (iii) simulations at the indicated CQ concentrations. (**C**) ERP restitution curves for the WT, WT-D172N (i), and D172N (ii) simulations at 3 µM concentration of CQ. (**D**) Measured slopes of ERP restitution curves for the WT (i), WT-D172N (ii), and D172N (iii) simulations at the indicated CQ concentrations.

The effects of CQ on APD prolongation of EPI cells was rate-dependent as shown by the APD restitution (APD-R) curves in Figure 4A for the WT and mutant WT-D172N (i) and D172N (ii) conditions. Over a range of diastolic intervals (DI) studied, the APD was prolonged in the mutant conditions with the actions of CQ. The APD-R relationship was also flattened by the action of CQ, as indicated by the decreased maximal slope for each APD-R curve in the WT, WT-D172N, and D172N simulations as shown in Figure 4Bi–iii, respectively. The ERP prolongation was also rate-dependent. It was prolonged under the CQ-in-action condition across a range of BCLs as shown in ERP restitution (ERP-R) curves in Figure 4C. Actions of CQ also flattened the ERP-R curves, as indicated by the decreased maximal slope of the ERP-R curves (Figure 4D). ERP is a measure of cell excitability and defines the minimum period allowable for the propagation of premature stimulus, indicating that CQ increased tissue’s excitability in SQT3. As a decreased steepness of APD-R and ERP-R curves are believed to be associated with stability of re-entry, inclining to terminate re-entrant excitation waves and to eliminate multiple re-entrant excitation wavelets. CQ also shifted the ERP-R curves of the WT-D172N and D172N conditions rightward, indicating that CQ did not enable ventricular cells to support higher rate electrical activity in SQT3 (as seen during ventricular fibrillation).

### Effects of chloroquine on SQT3 in a 1D fibre model

**Figure 5:**
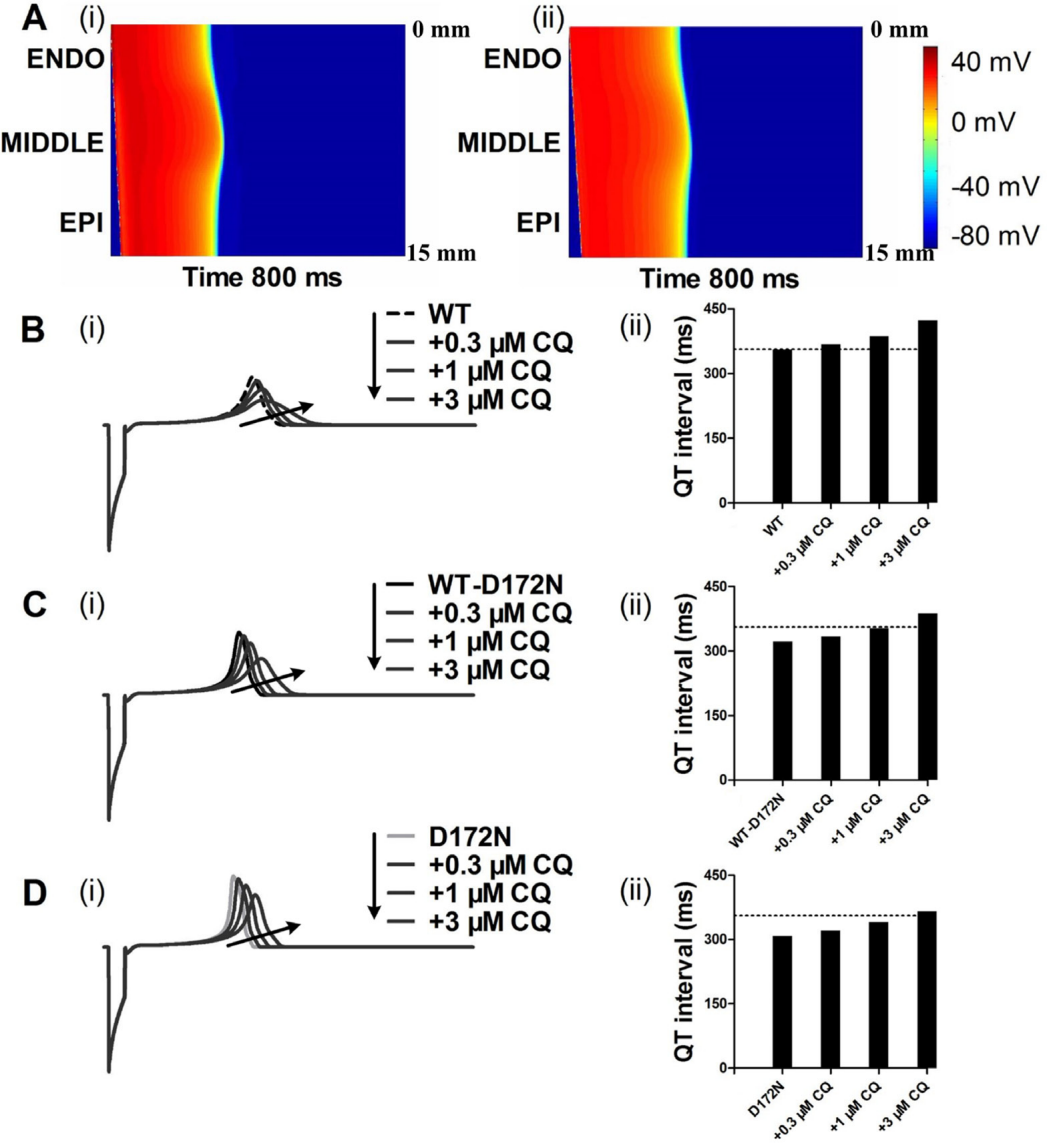
Dose-dependent effects of CQ on the pseudo-ECG of a 1D transmural strand in the WT, WT-D172N and D172N conditions (**A**) Excitation prolongation across a 1D transmural strand model for the WT-D172N (i) simulations at 3 µM concentration of CQ (ii). Space runs from the top (ENDO, 0 mm) to the bottom (EPI, 15 mm). Time runs from the left (0 ms) to the right (800 ms). The corresponding pseudo-ECGs derived from the propagating electrical excitation wave. (**B**) Superimposed pseudo-ECGs (i) for the WT simulations at the indicated concentrations and the corresponding QT interval histogram (ii). (**C**) Superimposed pseudo-ECGs (i) for the WT-D172N simulations at the indicated concentrations and the corresponding QT interval histogram (ii). (**D**) Superimposed pseudo-ECGs (i) for the WT simulations at the indicated concentrations and the corresponding QT interval histogram (ii).

Using a 1D fibre model of the ventricular wall, we computed a pseudo-ECG under the CQ-in-action condition (Figure 5). Pseudo-ECG traces were extracted from a propagating wave from the ENDO towards MIDDLE and EPI parts of the strand (Figure 5A). Pseudo-ECG traces were plotted for WT (Figure 5B), WT-D172N (Figure 5C) and D172N (Figure 5D) conditions at the indicated CQ concentrations. Consistently with AP simulations, a prolongation of the QT interval in the presence of CQ can be observed when the 1D model was used. The QT interval was prolonged from 322 ms in WT-D172N condition to 334, 353, and 388 ms in the presence of 0.3, 1, and 3 µM doses of CQ, respectively, and from 308 ms in the D172N condition to 321, 340, and 366 ms at the indicated CQ concentrations. The prolongation of QT intervals for the WT-D172N and D172N simulation at the 3 µM concentration of CQ was within the normal physiological range (from 360 to 440 ms). The shortening of the QT interval is accompanied by an increased T-wave amplitude, as observed in the previous studies [7, 10], in which the kinetics of the D172N mutation of the *KCNJ2* subunit of the *I*_K1_ channel was incorporated into the human ventricular AP model and pseudo-ECG simulations. When CQ at the indicated concentrations was used, the higher T-wave amplitude for the mutant WT-D172N and D172N conditions was decreased as also shown in Figure 5.

**Figure 6:**
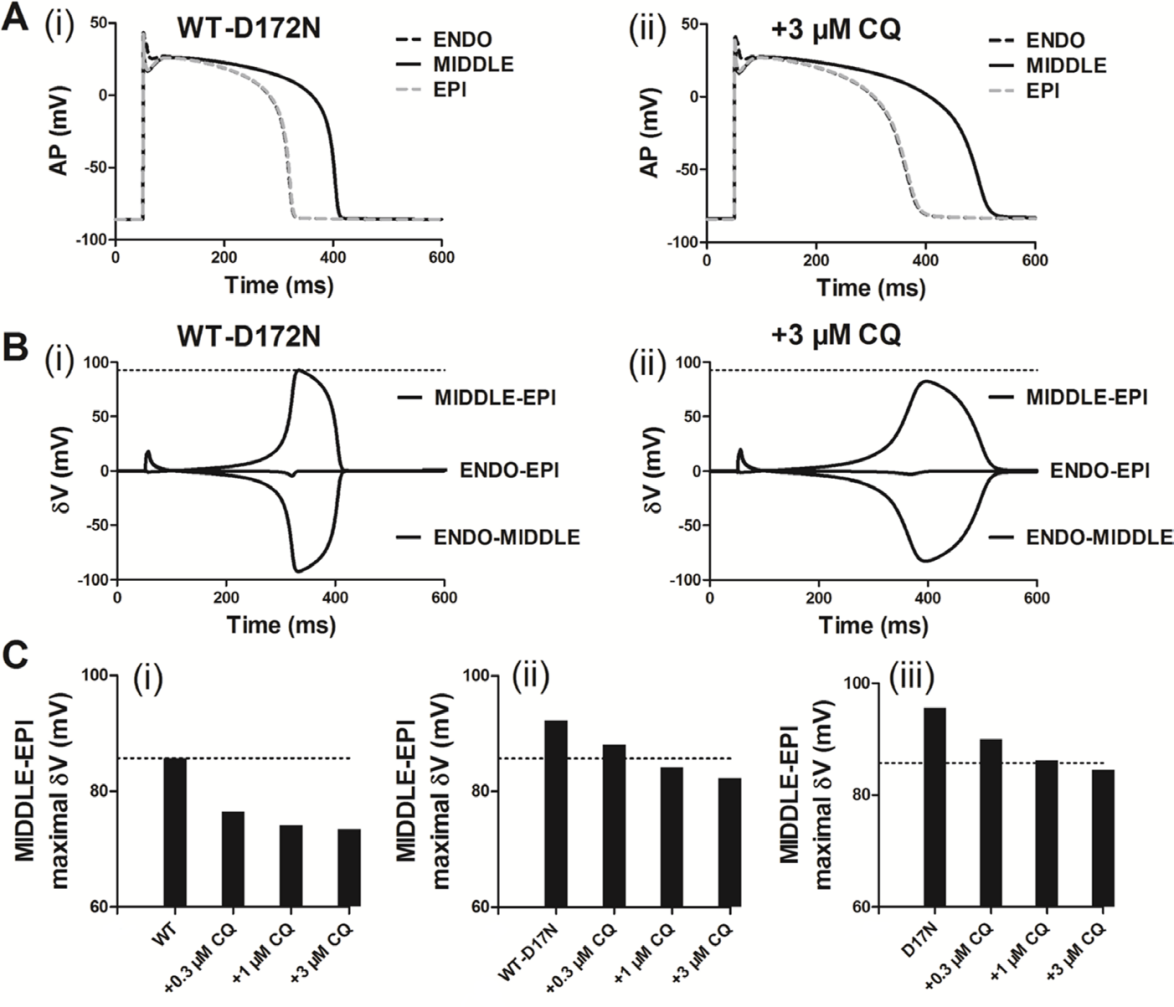
Membrane voltage heterogeneity (*δV*) between ENDO, MIDDLE, and EPI cells and transmural APD_90_ distribution and its spatial gradient in the 1D transmural strand (**A**) ENDO, MIDDLE, and EPI APs for the WT-D172N simulations (i) at 3 µM concentration of CQ (ii). (**B**) Plots of δV against time for the WT-D172N simulations (i) at 3 µM concentration of CQ (ii). (**C**) Maximal *δV* observed during repolarization (MIDDLE-EPI) for the WT (i), WT-D172N (ii) and D172N (iii) simulations at the indicated CQ concentrations.

**Figure 7:**
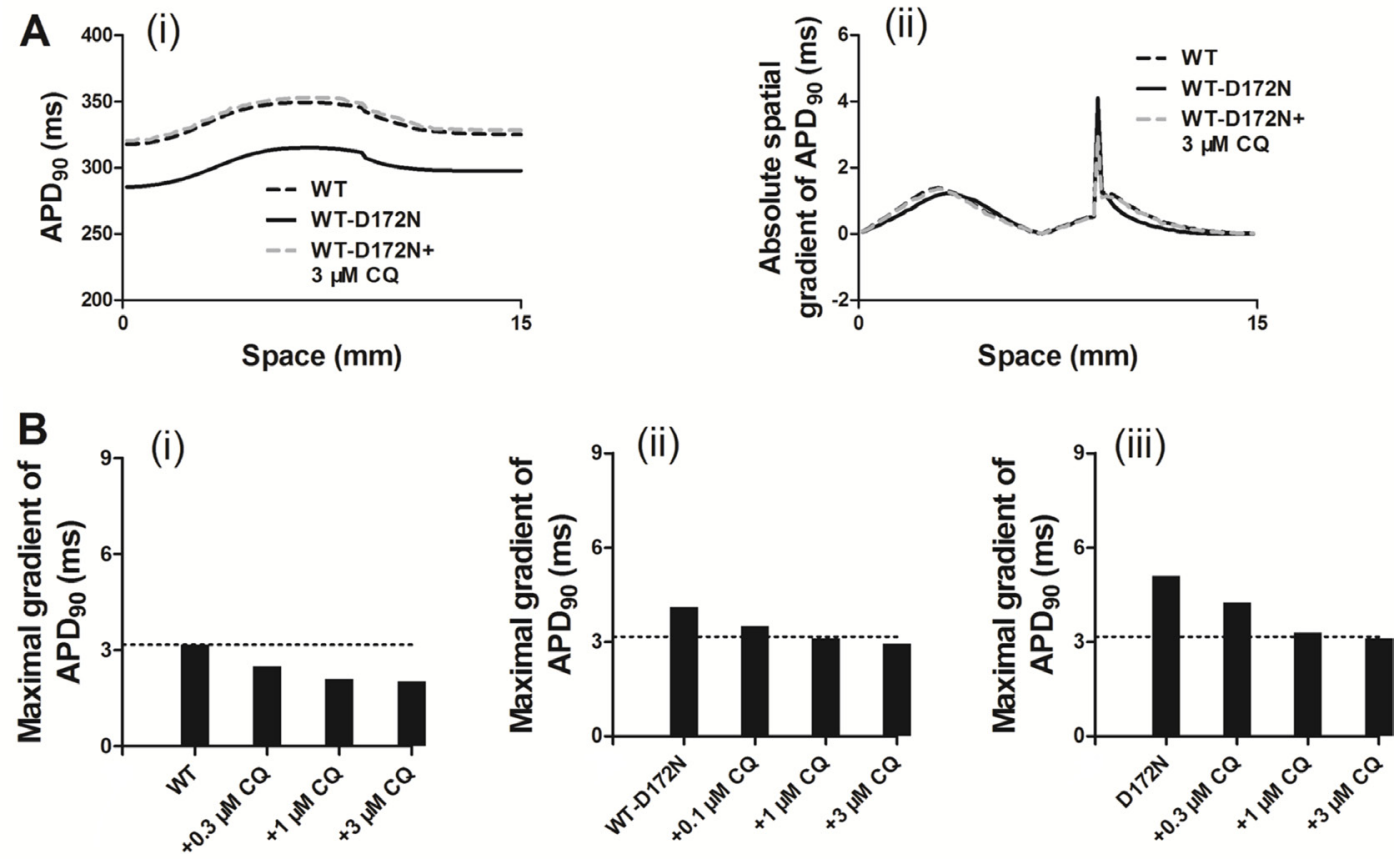
Spatial distribution of APD_90_ and its spatial gradient in a 1D transmural strand for the WT, WT-D172N, and D172N simulations at the indicated CQ concentrations (**A**) The spatial gradient of APD_90_ (i) and its spatial gradient (ii) in a 1D transmural strand. Space runs from the left (ENDO, 0 mm) to the right (EPI, 15 mm) on the *x*-axis. (**B**) The maximal spatial gradient of APD_90_ in a 1D transmural strand for the WT (i), WT-D172N, (ii) and D172N (iii) simulations at the indicated CQ concentrations.

Previous studies [10, 27, 28] showed that an increase in T-wave amplitude in SQTS was attributed to an increased spatial gradient of membrane potential, and an inverse effect might explain the decrease T-wave amplitude seen in Figure 5 of this study. Therefore, we examined the effects of CQ on membrane voltage heterogeneity (*δV*) between ENDO, MIDDLE, and EPI cells. Figure 6A shows the simulated ENDO, MIDDLE, and EPI APs for the WT-D172N (i) and WT-D172N + 3 µM CQ (ii) conditions while Figure 6B shows the corresponding time-course plots of the *δV*. In the presence of CQ condition, the maximal *δV* of MIDDLE-EPI APs was smaller than under the mutant condition, which contributed to the decreased T-wave amplitude (Figure 6C). In the 1D model, the cell-to-cell electronic interactions smoothed out the APD distribution as shown in Figure 7A(i) for both the WT and mutant simulations at the indicated CQ concentrations. CQ flattened APD dispersion across the strand as shown by the plotted APD absolute spatial gradient in Figure 7A(ii) and corresponding maximal spatial gradient in Figure 7B(i–iii), which also led to the decreased T-wave amplitude.

**Figure 8:**
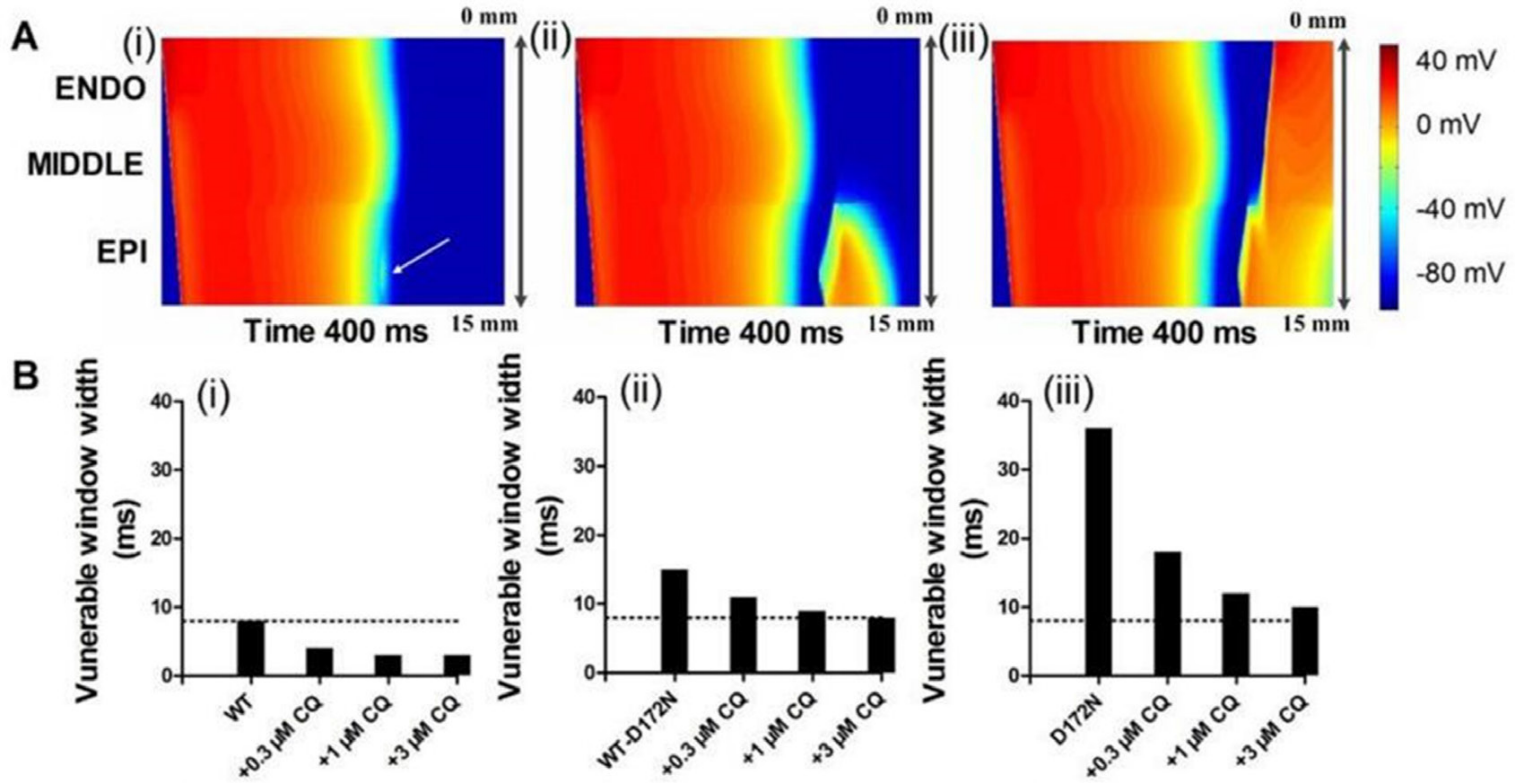
Simulated vulnerable window by using a 1D transmural strand model for the WT, WT-D172N, and D172N simulations at the indicated CQ concentrations (**A**) Space-time plot of excitation propagation and response of a 1D transmural strand to a test stimulus. APs are mapped into a colour spectrum ranging from –85 ms to 45 mV. Space runs from top (ENDO) to bottom (EPI). Time runs from left (0 ms) to the right (400 ms). The premature stimulus applied at EPI part at 320 ms, bi-directional block (i); 340 ms, unidirectional conduction block (ii); and 350 ms, bi-directional conduction (iii). (**B**) The vulnerability of the tissue for the WT (i), WT-D172N (ii), and D172N (iii) simulations at the indicated CQ concentrations.

In further simulations using the 1D fibre model, we measured the temporal vulnerability of the WT, WT-D172N, D172N, and CQ-in-action tissue to unidirectional block. Figure 8A shows the conditioning excitation wave and the response of the tissue to a test stimulus applied at EPI part of the strand (marked by arrow). In Figure 8A(i), the test stimulus was applied early (at time = 320 ms) so that the tissue did not have enough time to recover. As a consequence, a bidirectional conduction block was observed. In Figure 8A(ii), the test stimulus was applied within the vulnerable window (at time = 340 ms) that produced a unidirectional conduction block. In Figure 8A(iii), the test stimulus was applied after the vulnerable window (at time = 350 ms) and, consequently, a bi-directional conduction was observed. Figure 8B shows the width of the vulnerable window for the WT, WT-D172N, and D172N simulations at the indicated CQ concentrations. These simulation results show clearly that the temporal vulnerability in SQT3 is decreased by CQ.

### Effects of chloroquine on SQT3 in a 2D tissue model

**Figure 9:**
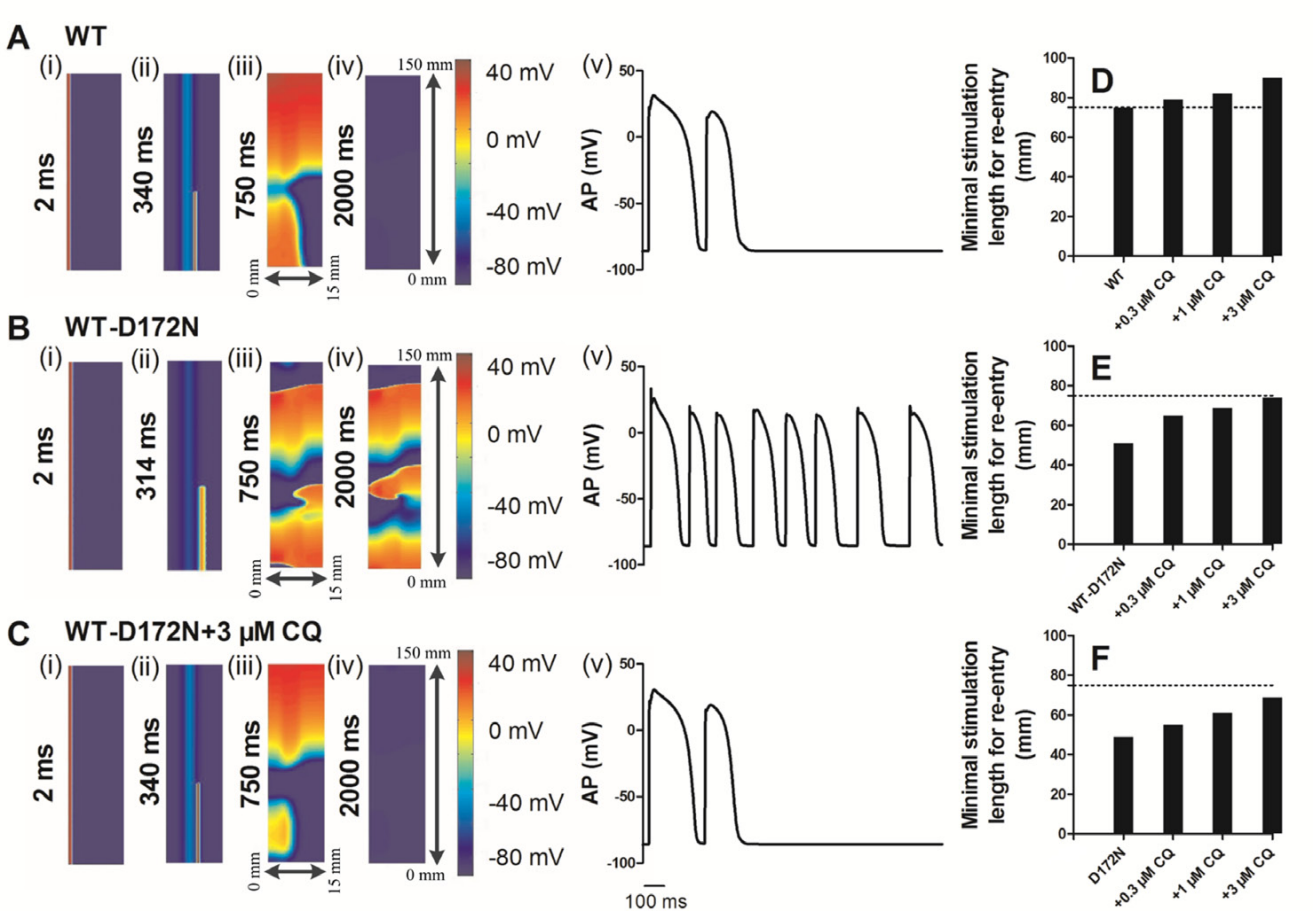
Snapshots of initiation and conduction of re-entry in a 2D idealized model of transmural ventricle for the WT, WT-D172N, and D172N simulations at the indicated CQ concentrations (**A**) In the WT condition, a planar conditioning wave generated by S1 stimulus at the ENDO end, which propagates through MIDDLE then towards the EPI end. An S2 stimulus applied to the MIDDLE-EPI junction during the vulnerable window of the local tissue. Space runs from the left (ENDO, 0 mm) to the right (EPI, 15 mm) on the *x*-axis. Space runs from the bottom (0 mm) to the top (150 mm) on the *y*-axis. Snapshots at time=2 ms (i), 340 ms (ii), 750 ms (iii), 2000 ms (iv) and the corresponding evolution of an EPI cell AP (v). (**B**) In the WT-D172N condition, a re-entry was initiated and maintained. Snapshots at time=2 ms (i), 314 ms (ii), 750 ms (iii), 2000 ms (iv) and the corresponding evolution of an EPI cell AP (v). (**C**) In the WT-D172N + 3 µM CQ condition, a re-entry was initiated and maintained. Snapshots at time=2 ms (i), 340 ms (ii), 750 ms (iii), 2000 ms (iv) and the corresponding evolution of an EPI cell AP (v). Spiral wave self-terminated in the WT condition before this recording point, but persisted in the WT-D172N condition. CQ prevented the spiral wave in the WT-D172N condition. (**D**, **E**, and **F**) Measured minimum spatial length of a premature S2 for the WT, WT-D172N, and D172N simulations at the indicated CQ concentrations.

In a 2D idealized human ventricular tissue, we investigated the pharmacological effects of CQ on re-entrant excitation waves. The results of 2D idealized tissue simulations are shown in Figure 9 (and Supplementary Videos 1–3). In simulations, an S1 was applied to the ENDO side to evoke a planar wave that propagated towards the EPI regions for the WT (Figure 9A(i)), WT-D172N (Figure 9B(i)) and WT-D172N + 3 µM CQ (Figure 9C(i)) conditions. After a time delay, a premature S2 was applied to the MIDDLE-EPI junction (Figure 9A(ii) for WT, Figure 9B(ii) for the WT-D172N and Figure 9C(iii) for the WT-D172N + 3 µM CQ), which produced a unidirectional conduction towards the EPI side due to a longer refractory period of the MIDDLE, forming a re-entry (Figure 9A(iii) for the WT, Figure 9B(iii) for the WT-D172N and Figure 9C(iv) for the WT-D172N + 3 µM CQ). In the WT condition (Figure 9A(iv)), the initiated re-entry was unstable with its tip meandering in a large area; this led to self-termination when the tip meandered out of the boundary of the tissue, but it was sustained for the WT-D172N condition (Figure 9B(iv)) (data of D172N condition not shown). With the use of CQ, re-entrant excitation wave in SQT3 terminated as shown in Figure 9C(iv). The time course of an EPI cell AP in the 2D idealized tissue is shown model for the WT, WT-D172N, and WT-D172N + 3 µM CQ conditions in Figure 9A(v), 9B(v), and 9C(v), respectively.

In these conditions, the formation of the induced re-entrant excitation wave was dependent on the spatial size of the premature stimulus. Therefore, we measured the minimal spatial size of a premature stimulus that enabled the formation of re-entry. The results are shown in Figure 9D–9F. CQ increased the minimum length of S2 stimulus in SQT3, and the re-entrant wave was found to be terminated. As the minimum substrate size quantifies (in a reciprocal fashion) the tissue’s spatial vulnerability, this increase demonstrated a reduced tissue’s susceptibility to arrhythmia in SQT3 by use of CQ.

**Figure 10:**
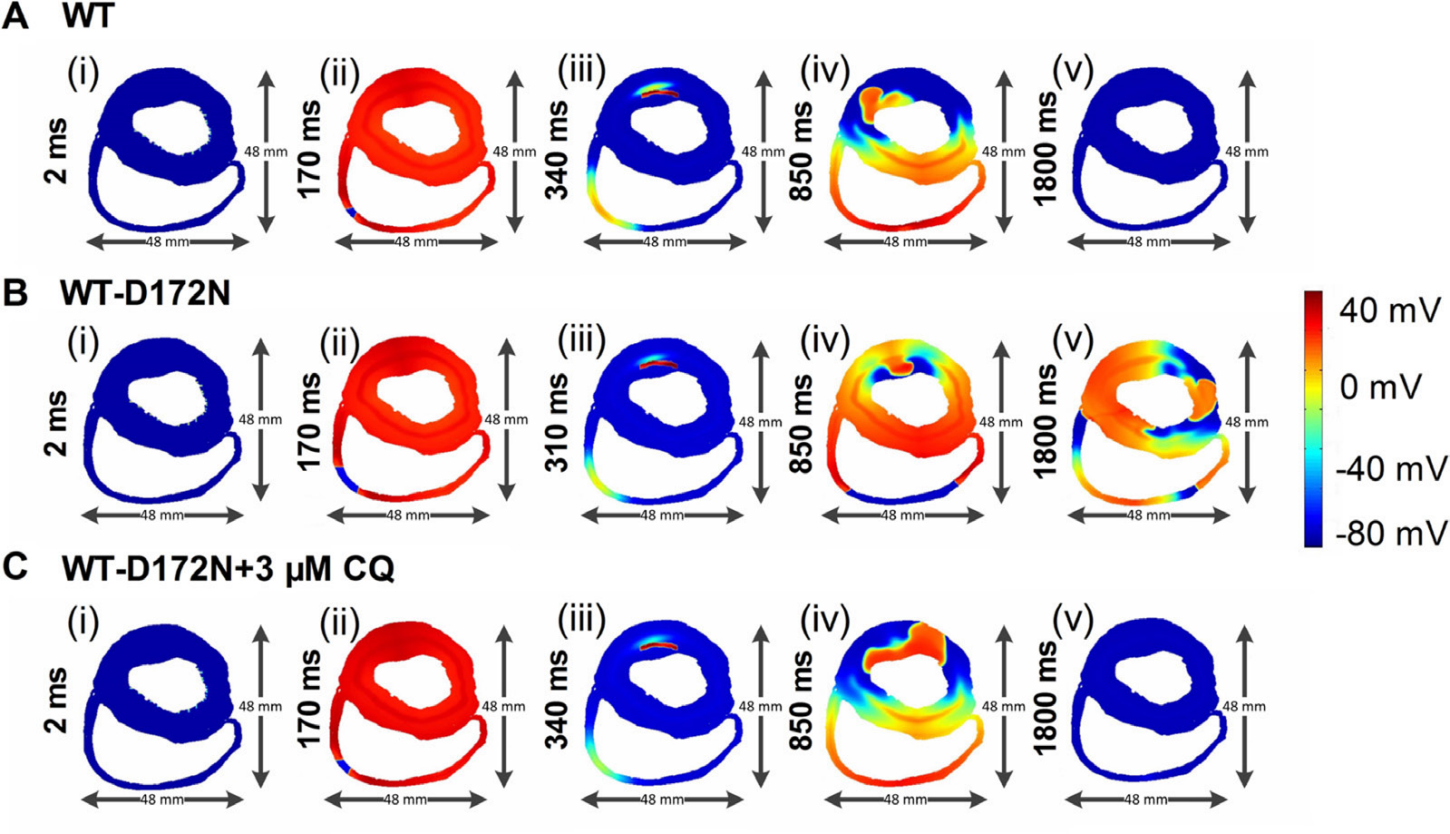
Snapshots of initiation and conduction of re-entry in the 2D slice model of human ventricles (**A**) In the WT condition, a conditioning wave generated by an S1 stimulus. Application of a premature S2 stimulus into the refractory and partially recovered region of an excitation wave after a delay of 340 ms. Snapshots at time = 2 ms (i), 170 ms (ii), 340 ms (iii), 850 ms (iv), and 1800 ms (v). (**B**) In the WT-D172N condition, a re-entry was initiated and maintained. Snapshots at time = 2 ms (i), 170 ms (ii), 310 ms (iii), 850 ms (iv), and 1800 ms (v). (**C**) In the WT-D172N + 3 µM CQ condition, a re-entry terminated. Snapshots at time = 2 ms (i), 170 ms (ii), 340 ms (iii), 850 ms (iv), and 1800 ms (v). Re-entrant wave self-terminated in the WT condition before this recording point but persisted in the WT-D172N condition. CQ eliminated the wavelets and terminated the re-entrant wave.

Due to the complex properties of ventricular tissue, it cannot be assumed that terminated re-entry in a 2D idealized tissue model necessarily translates into similar activity in a more realistic model. Therefore, further simulations were performed in a human ventricle cross-sectional slice. Results of the 2D slice are shown in Figure 10 (and Supplementary Videos 4–6). Figure 10 shows subsequent conduction of the induced re-entry from the applied premature S2 for the WT (Figure 10A), WT-D172N (Figure 10B), and WT-D172N + 3 µM CQ (Figure 10C) conditions. In the WT condition, the initiated re-entrant excitation wave was unstable, with its tip meandering around in the tissue, leading to self-termination when it meandered out of the boundary of the tissue. However, in the WT-D172N condition, the tip of the reentrant wave also meandered, but in a small region when compared with the WT condition. With the use of CQ in the WT-D172N condition, the re-entrant wave was terminated by meandering out of tissue border.

### Effects of chloroquine on SQT3 in a 3D anatomical model

**Figure 11:**
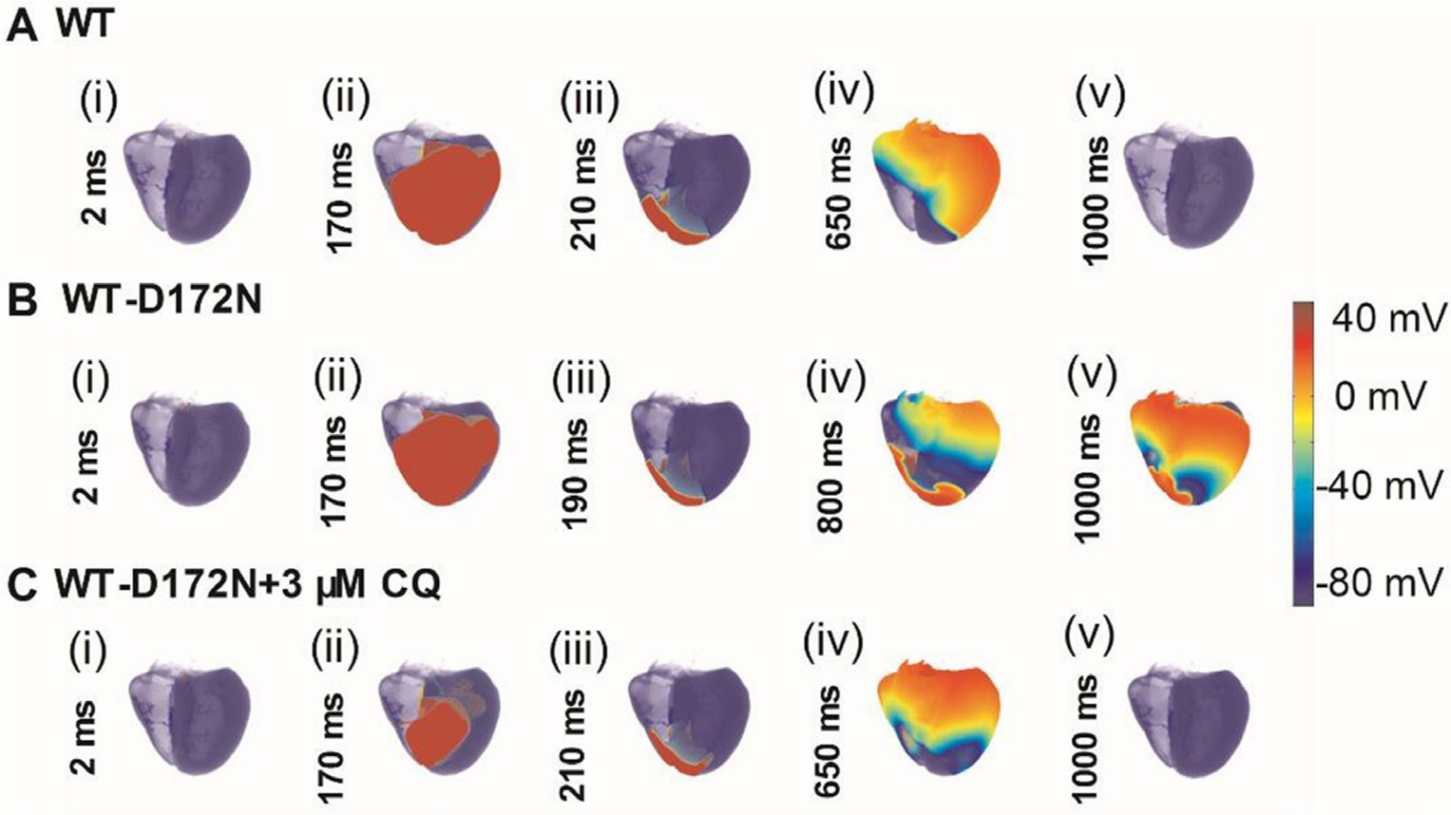
Snapshots of initiation and conduction of re-entry in the 3D model of human ventricles (**A**) A conditioning wave generated by an S1 stimulus. Scroll wave was initiated by using the cut-wavefront protocol after a delay of 210 ms for the WT condition from the initial wave stimulus. Snapshots at time = 2 ms (i), 170 ms (ii), 210 ms (iii), 650 ms (iv), and 1000 ms (v). (**B**) Scroll wave was initiated by using the cut-wavefront protocol after a delay of 190 ms for WT condition from the initial wave stimulus. Snapshots at time = 2 ms (i), 170 ms (ii), 190 ms (iii), 800 ms (iv), and 1000 ms (v). (**C**) Scroll wave was initiated by using the cut-wavefront protocol after a delay of 210 ms for the WT condition from the initial wave stimulus. Snapshots at time = 2 ms (i), 170 ms (ii), 210 ms (iii), 650 ms (iv), and 1000 ms (v). Scroll wave self-terminated in the WT condition before this recording point, but persisted in the WT-D172N condition. CQ terminated the scroll wave.

We performed further simulations with a 3D anatomical human ventricle geometry. The results are shown in Figure 11 (and Supplementary Videos 7–9), which show the snapshots of the evolution of re-entrant scroll waves (WT: Figure 11A(i–v); WT-D172N: Figure 11B(i–v); WT-D172N + 3 µM CQ: Figure 11C(i–v)) by using the cut-wavefront protocol after a delay of the initial excitation wave. In the WT condition, the scroll wave was unstable and non-stationary, leading to self-termination. However, under the heterozygous WT-D172N condition, the scroll wave was sustained. When in the presence of CQ condition, the scroll wave became unstable and terminated. The 3D results are consistent with the results shown in Figures 9 and 10 with 2D results, thereby further supporting the notion that the actions of CQ decrease tissue susceptibility to arrhythmogenesis by terminating re-entrant excitation waves.

## DISCUSSION

### Summary of major findings

Possibly due to a lack of phenotypically accurate experimental models, there has hitherto not been any detailed investigation of how the anti-arrhythmic drug influences ventricular cellular and tissue electrophysiology in SQTS. In the present study, we adopted a computational approach to gain potential pharmacological effects of CQ on SQT3.

Our main findings are summarised as follows: (i) the modified *I*_K1_ formulations reproduce the dynamic properties of *I*_K1_ and clinically-relevant characteristics of SQT3 (including markedly short QT intervals and higher T-wave amplitude); (ii) CQ at the indicated concentrations prolongs the APD_90_ and ERP, and attenuates the APD-R and ERP-R curves, thus decreasing susceptibility to arrhythmia; (iii) CQ prolongs the QT intervals and reduces the T-wave amplitude, which is within the normal physiological range; (iv) decreases the maximal voltage heterogeneity (*δV*) between ENDO, MIDDLE, and EPI cell APs, and decreases the maximal dispersion of APD_90_, which subsequently leads to the decreased T-wave amplitude; (v) CQ reduces the tissue’s temporal vulnerability to the genesis of unidirectional conduction by a premature excitation, and increases the minimal substrate size of tissue required to maintain re-entry (spatial vulnerability), such that the overall susceptibility of tissue is reduced; (vi) CQ terminates re-entrant excitation waves as shown in both 2D and 3D models of the human ventricles. Collectively, these findings provide mechanistic insight into the anti-arrhythmic effects of CQ on SQT3 arising from *KCNJ2* D172N mutation, in terms of propensity for termination of re-entrant excitation waves.

### Significance of the study

Previous studies [10, 27, 28, 30, 31] have used mathematical models to explore the functional impact of the SQT1, SQT2, and SQT3 gene mutations on ventricular cell AP shortening and characteristics of pseudo-ECGs. However, while investigations of the pro-arrhythmic effects of the *KCNJ2* D172N mutation (SQT3) on perpetuating re-entrant excitation waves in ventricular tissue have been conducted before, simulations addressing the pharmacological effects of drugs on SQT3 have not been performed until now. Furthermore, the present study is the first to determine the effects of anti-malarial drug CQ on SQT3 by using multi-scale models of the human ventricles.

Our data constitute evidence that the anti-arrhythmic effects of CQ on tissue vulnerability to the initiation and maintenance of re-entry. In this study, tissue susceptibility was indexed by its temporal and spatial vulnerability. This provides a means for reduced tissue’s susceptibility to re-entry in SQT3 with the use of CQ. CQ prolonged ventricular repolarization, which resulted in a decreased temporal vulnerability (the width of vulnerable time window). Spatial vulnerability to arrhythmia is determined by the (reciprocal relationship) critical tissue size required to accommodate the pathway of re-entry and thereby enable re-entrant excitation waves to become sustained. This index of arrhythmia susceptibility is related to the wavelength of the excitation wave, defined as the product of ERP and CV. Therefore, a prolongation of ERP due to CQ causes an increased wavelength of excitation, required a bigger tissue substrate size to enable re-entry to become sustained. Thus, re-entry in SQT3 at the indicated CQ concentrations self-terminated shortly after initiation in 2D and 3D models. This is due to the increased ERP and thus an increased wavelength of re-entry, which is not sustained in a limited mass of tissue. Our findings also indicate that CQ reduces transmural heterogeneity of APD. This led to a reduced maximal APD dispersion in the transmural strand. This in turn is anti-arrhythmic as it reduced the tissue’s vulnerable time window.

The simulation results in this study indicate CQ concentration 3 µM is sufficient to normalize SQT3 condition. Interestingly, plasma concentrations are in the range of 2–3 µM following administration of therapeutic doses of CQ [32]. Thus, considered collectively, our simulation results provide evidence that CQ at therapeutic concentrations may be able to cure patients with SQT3.

### Relevance to previous studies

Abnormality of Kir2.1 channels arising from *KCNJ2* mutations has been identified in various cardiac diseases. A loss-of-function mutation in gene *KCNJ2* encoding Kir2.1 causes Andersen-Tawil Syndrome, an autosomal dominant disorder characterized by QT interval prolongation and ventricular arrhythmia [33, 34]. A gain-of-function mutation in gene *KCNJ2* causes one form of SQTS (SQT3) [7]. Patients with this mutation that augmented *I*_K1_ exhibit shortened APD and ERP, and increased susceptibility to arrhythmias. We investigated that augmented *I*_K1_ due to the *KCNJ2* D172N mutation increased susceptibility to re-entry and perpetuation of re-entrant arrhythmia in SQT3 [10]. Previous studies [35, 36] have reported that blockade of *I*_K1_ terminated VF in guinea pig heart and it may offer a potential therapeutic target for cardiac arrhythmia treatment. Significantly, anti-malarial drug CQ has been shown previously to block preferentially outward over current for both *I*_Kir2.1_ and *I*_K1_ [16, 37], and this would appear well-suited to reducing consequences of a gain-of-function mutation to Kir2.1. CQ was identified as an effective inhibitor of SQT3 mutant Kir2.1 channels and suggested CQ might lengthen cardiac repolarization in SQT3 [14, 15]. In the present study, our data indicate that with the use of CQ, the resulting decrease in outward *I*_K1_ not only helps to terminate re-entry but also reduces the susceptibility to arrhythmogenesis.

### Potential limitations of the study

The ten Tusscher *et al.* model was used here to simulate the cellular electrical activity of human ventricular myocytes, and its limitations have been discussed in detail elsewhere [10, 27–29, 38, 39]. Limitations of 1D-3D models of the human ventricles have been discussed in our previous work [10, 27, 28]. For example, tissue models in this study do not consider the effects of cardiac mechanics on tissue geometry, which feasibly might influence re-entry.

Another limitation is the use of the animal model to mathematically describe the effects of CQ on the ionic currents as listed in Table 1. Although animal models have contributed much to our understanding of mechanisms of human diseases, their value in predicting the effectiveness of pharmacological treatment has remained controversial [40, 41]. For future work, integration of experimental data for the different ion channels of the human heart when becoming available could provide a better insight into the pharmacological effects of CQ on SQT3.

**Table 1:** Concentration-dependent block effects of CQ on different ionic currents and conductivities (% of original value)

Conditions	Current /Conductivity	CQ concentration (µM)			Source
		0.3	1	3	
WT	*I*_K1_/*G*_K1_	12.5%	41.6%	75.1%	[14]
	*I*_Kr_/*G*_Kr_	21%	38%	65%	[16]
WT-D172N	*I*_K1_/*G*_K1_	14.7%	40%	69.4%	[14]
	*I*_Kr_/*G*_Kr_	21%	38%	65%	[16]
D172N	*I*_K1_/*G*_K1_	17.8%	45.4%	35%	[14]
	*I*_Kr_/*G*_Kr_	21%	38%	65%	[16]

Nevertheless, while it is useful to make these potential limitations of the present study explicit, these are not anticipated to influence fundamentally the conclusions that can be drawn on likely mechanisms by which the actions of CQ have anti-arrhythmic effects on SQT3.

## CONCLUSIONS

On the basis of this simulation study, it can be concluded that the CQ causes the prolongation of QT interval and it reduces the maximal dispersion of APD and difference in membrane potential (*δV*) during APs that reduce tissue vulnerability to arrhythmogenesis. Moreover, CQ prolongs ventricular tissue ERP that terminates the re-entry in both 2D and 3D tissue scenarios. In conclusion, the findings constitute new evidence that the anti-arrhythmic effects of CQ on SQT3 and, by extension, to the possibility that CQ may be a potential therapeutic agent for SQT3 treatment. Additionally, the multi-scale human ventricular models employed in this study may have further utility for probing the effects of other drugs on SQT3 and other forms of SQTS.

## MATERIALS AND METHODS

### The model of the human ventricle and *I*_K1_ kinetics

To date, there are three principal models that describe the time- and voltage-dependent ionic currents, and reconstruct the AP of human ventricular cells – ten Tusscher *et al.* model [29], Grandi *et al.* model [42], and ORd *et al.* model [43]. All these AP models were mathematically reconstructed from ionic processes that were formulated by available data obtained from human beings. The ten Tusscher model has been found to be well-suited to the study of reentrant arrhythmias in human ventricles and therefore was used in this study. Cell computations presented here were coded in C++ format and integrated using the forward Euler method with a fixed time step of 0.02 ms.

We changed the parameters in the equations for *I*_K1_ in agreement with experimentally obtained current-voltage (I–V) data of Kir2.1 current to simulate the mutant conditions (including heterozygous WT-D172N and homozygous D172N mutant scenarios) [15]. This was achieved by simulating the previous experimental voltage-clamp protocol data [15], with which the experimental data were fitted to model equations by the Broyden-Fletcher-Goldfarb-Shanno optimization algorithm [10]. Relative current proportions were scaled by using relative proportions of peak *I*_Kir2.1_; 2.2-fold for WT-D172N condition and 4.6-fold for the D172N condition than for WT condition [10, 15]. The modified equations and parameters of *I*_K1_ [10] are provided in the Supplementary Information.

The *I*_K1_ formulations were incorporated into the chosen human ventricular cell model. The modified cell model was then incorporated into 1D, 2D and 3D models of human ventricle based on a partial differential equation with the form:

$$C_{m}\frac{\partial V}{\partial t}=-\left(I_{ion}+I_{stim}\right)+\nabla \cdot \left(D\nabla V\right)$$ (1)

where *D* is the diffusion coefficient determined by gap junction resistance, *t* is time, *I*_stim_ is the additionally applied stimulus current, *C*_m_ is the membrane capacitance per unit surface area, and *I*_ion_ is the sum of all ionic currents flowing through the cell membrane. The component of the late sodium current (*I*_NaL_) from the ORd model [43] was incorporated. In simulations, gap junction conductance was set to a constant value of 0.0008 cm^2^/ms, which promoted an electrical conduction velocity (CV) of 52 cm/s, close to the experimental data of ∼50 cm/s [44, 45]. Gap junction conductance was set homogeneous, except for a 5-fold decrease at the MIDDLE-EPI transition border. This followed the approach of Gima and Rudy [46] (and our previous studies on SQT1-3 [10, 27, 28]).

For 1D computation, AP propagation was reconstructed as described previously in a 1D fibre [10, 27, 28, 46]. This reconstruction reproduces the initiation of APs that propagate from ENDO- to EPI-region during physiological ventricular excitation. The 1D fibre (15 mm length) composed by 25 ENDO, 35 MIDDLE, and 40 EPI cells has been considered. These proportions were similar to those used in other studies [10, 27, 28, 30, 31, 46]. 2D models are used to quantify the initiation and maintenance of re-entrant excitation waves. In 2D simulations, both idealized and slice sheet geometries were implemented. The idealized geometry was a simple sheet of tissue measuring 15 mm by 150 mm. It was modelled by expanding the 1D fibre (length of 15 mm in the *x*-direction) into a sheet with a width of 150 mm in the y-direction. The slice geometry was a transverse cross-section slice which was composed 33125 ventricular cells with a spatial resolution of 0.2 mm. The 2D model of a slice through ventricles was segmented into distinctive regions of ENDO, MIDDLE, and EPI layers. It implemented anisotropic fibre orientations as used in the previous study by our group [10]. The intercellular conductivities in the fibre (transmural strand) and cross-fibre directions were set to 0.0024 and 0.0008 cm^2^/ms respectively. 3D models represent anatomical organs. A 3D model of human ventricles including 6941468 ventricular cells was based on the Visible Human Project (http://www.nlm.nih.gov/research/visible/visible_human.html). Details of the 3D model used are documented in previous studies [10, 27, 47, 48].

Details regarding methods for computing pseudo-ECG, protocols used for action potential duration (APD), effective refractory period (ERP), vulnerability of ventricular tissue, initiation of re-entry, and numerical methods have been documented in our previous studies [10, 26–28] and are provided in the Supplementary materials.

### Modelling drug/ion channel binding interactions

We analyzed the pharmacological effects of drug/ion channel binding interactions by using a simple pore block theory [49] as done by other groups, such as Y. Rudy [50], B. Rodriguez [49], and G. Seemann [51, 52]. In this study, the fractional block of ionic currents due to CQ was modelled using a blocking factor *θ*:

$$\theta =\frac{1}{1+\frac{IC_{50}}{{\left[CQ\right]}^{nH}}}$$ (2)

where [*CQ*] is the concentration, IC_50_ is the half-maximal inhibitory concentration, and nH is Hill coefficient. CQ primarily blocks the *I*_K1_ and the *I*_Kr_. From the experimental data [14], IC_50_ is 1.4 ± 0.1 µM with a nH of 1.3 for the WT condition, 1.5 ± 0.2 µM with a nH of 1.1 for the WT-D172N condition and 1.2 ± 0.1 µM with a nH of 1.1 for the WT-D172N condition (*n* = 5 cells). CQ inhibits the peak *I*_K1_ current in a concentration-dependent manner, and the potency of CQ block is qualitatively similar for the WT, WT-D172N and D172N conditions [14, 15]. In this study, several doses (0.3, 1 and 3 µM) were selected to assess the effects of CQ on SQT3. The resulting ion channel conductivity relative to their original values in the presence of CQ are provided in Table 1.

## SUPPLEMENTARY MATERIALS FIGURES AND VIDEOS





















## Abbreviations

(SQTS): Short QT syndrome
(SCD): sudden cardiac death
(ECG): electrocardiogram
(*I*_K1_): inward rectifier K+ current
(AP): action potential
(APD): action potential duration
(ERP): effective refractory period
(ICD): implantable cardioverter defibrillator
(*I*_Kr_): rapid delayed rectifier K+ current
(RP): resting potential
(APD-R): APD restitution
(DI): diastolic intervals
(ERP-R): ERP restitution
(δV): membrane voltage heterogeneity
(I-V): current-voltage
(*I*_NaL_): late sodium current
(CV): conduction velocity
