# Supplementary material for: Modelling the effects of chloroquine on KCNJ2-linked short QT syndrome
Source: Oncotarget. 2017 Nov 18;8(63):106511–26. doi: 10.18632/oncotarget.22490 (PMC5739752; doi:10.18632/oncotarget.22490)
Supplement: Supplementary file 1 [file oncotarget-08-106511-s001.pdf]

Modelling the effects of chloroquine on *KCNJ2*-linked short QT syndrome

SUPPLEMENTARY MATERIALS

Measurement of APD

Action potential duration (APD) was computed from single cell models by using an S1-S2 pacing protocol consisting of 10 conditioning stimuli (S1) (during which the model reached a stable solution) and a premature stimulus (S2) [1, 2]. S1 was applied at a frequency of 1.25 Hz. S2 was applied with variable diastolic intervals (DI) after the AP evoked by the last S1. S1 and S2 have the same strength and duration (–52 pA/pF and 1 ms). APD at 90% repolarization (APD_{90}) of APs were measured. APD restitution (APD-R) curve was computed as the derivate of APD_{90} evoked by the S2 against the DIs.

Measurement of ERP

Effective refractory period (ERP) was computed from single cell models by using a slightly different S1-S2 pacing protocol with 10 conditioning stimuli (S1) at a variable basic cycle length (BCL) followed by a premature stimulus (S2) with variable time delays (Δt). ERP was measured as the minimal Δt for which the S2 produced a peak voltage over 80% of the AP peak value evoked by the last S1 [1, 2]. S1 and S2 have the same strength and duration (–52 pA/pF and 1 ms). ERP restitution (ERP-R) curve was generated by the ERP against BCLs [3].

Computing the pseudo-ECG

The pseudo-ECG was computed by using Gima and Rudy method [4]:

$$\phi_e(x') = \frac{\alpha^2}{4}\int(-\nabla V)\cdot[\nabla\frac{1}{r}]dx \qquad (1)$$

where $Ø_e$ is the computed potential at the electrode, α is the radius of the strand, r is the Euclidean distance from a source point x to the electrode point x', and dx is the spatial resolution. The virtual electrode was placed at a position 2.0 cm away from the ENDO end of the fiber. The QT interval was calculated as the time interval determined by definition of the Q-wave onset (0.0 mV) and the point corresponding to the T-wave end (T_{end}). The time, at which the ECG data fell below a threshold (0.01 mV) was defined as T_{end}.

Measurement of temporal vulnerability of ventricle

In cardiac tissue (Supplementary Figure 1A), a propagating wavefront is followed by a refractory tail. A stimulus applied too late after the refractory tail will lead to excitation that propagates in both directions (bi-directional conduction) or if applied too early, excitation propagation that fails in both directions (bi-directional block). During the refractory tail, there is a time window, the vulnerable window (VW), during which stimulation produces a solitary wave that propagates in either the retrograde or anterograde direction, resulting in a uni-directional block that allows re-entry [1, 2, 5]. An S1-S2 protocol was used to investigate this. 10 S1 stimuli (1.25 Hz, strength: –52 pA/pF, duration: 1 ms) was applied to evoke a wave. Following a time delay after the 10th stimulus, an S2 (with the same of stimulus strength and duration as the S1) was applied to the marked region of the stand (marked with an arrow in Figure 8). The width of the time window during which the S2 propagated uni-directionally in the strand was measured as the temporal vulnerability of ventricular tissue.

Measurement of critical size of ventricular tissue to support re-entry – spatial vulnerability

In order to measure the critical substrate size of ventricular tissue (Supplementary Figure 1B) required to sustain re-entry, a standard S1-S2 protocol was used [1]. An S1 was applied to ENDO edge of the 2D idealized tissue to evoke a conditioning planar wave. After a time delay, an S2 was applied to the MIDDLE-EPI junction in the refractory tail of the conditioning wave. If the length of S2 is sufficiently long, the tip has sufficient space to follow its circuit path, and the re-entry survives. However, if the length of S2 is short, there is insufficient space for the tip to follow the path. Then the tip meanders out of the boundary of the tissue and wave terminates. The critical size of the tissue to support re-entry was quantified as the minimal length of S2 stimulus that supports the initiation

and maintenance of re-entry, which provides an indication of the susceptibility of tissue to re-entry: the larger the critical size, the more difficult the initiation of re-entry in the tissue [1].

List of model equations and parameters

The inward rectifier K^+ current (I_{K1}) was modified based on the experimentally obtained data [6], which are described previously [7]:

$$I_{K1} = G_{K1}\sqrt{\frac{Ko}{5.4}}x_{K1\infty}(V - E_K) \tag{2}$$

$$x_{K1\infty} = \frac{\alpha_{K1}}{\alpha_{K1} + \beta_{K1}} \tag{3}$$

WT:

$$\alpha_{K1} = \frac{0.07}{1 + e^{0.017(V - E_K - 200.2)}} \tag{4}$$

$$\beta_{K1} = \frac{3e^{0.0003(V - E_K + 100.2)} + e^{0.08(V - E_K - 8.7)}}{1 + e^{-0.024(V - E_K)}} \tag{5}$$

$$G_{K1} = 4.8\frac{nS}{pF} \tag{6}$$

WT-D172N:

$$\alpha_{K1} = \frac{0.1}{1 + e^{0.023(V - E_K - 199.9)}} \tag{7}$$

$$\beta_{K1} = \frac{3e^{0.0002(V - E_K + 100.4)} + e^{0.07(V - E_K - 9.8)}}{1 + e^{-0.021(V - E_K)}} \tag{8}$$

$$G_{K1} = 6.27\frac{nS}{pF} \tag{9}$$

D172N:

$$\alpha_{K1} = \frac{0.1}{1 + e^{0.05(V - E_K - 199.9)}} \tag{10}$$

$$\beta_{K1} = \frac{3e^{0.0002(V - E_K + 100.1)} + e^{0.08(V - E_K - 10.3)}}{1 + e^{-0.006(V - E_K)}} \tag{11}$$

$$G_{K1} = 11.32\frac{nS}{pF} \tag{12}$$

where G_{K1} is the maximal conductance.

Model independence of the effects of CQ on SQT3

In addition to using the ten Tusscher and Panfilov ventricular cell model [8], we also employed the ORd human ventricular cell model [9] to predict the effects of CQ on SQT3. The data obtained with this model support our original results; APD was prolonged due to the presence of CQ (Supplementary Figure 2). Consequently, the effects of CQ on SQT3 are model independent.

REFERENCES

1. Zhang H, Kharche S, Holden AV, Hancox JC. Repolarisation and vulnerability to re-entry in the human heart with short QT syndrome arising from KCNQ1 mutation—a simulation study. Prog Biophys Mol Biol. 2008; 96:112–31. https://doi.org/10.1016/j.pbiomolbio.2007.07.020.
2. Kharche S, Garratt CJ, Boyett MR, Inada S, Holden AV, Hancox JC, Zhang H. Atrial proarrhythmia due to increased inward rectifier current (I(K1)) arising from KCNJ2 mutation—a simulation study. Prog Biophys Mol Biol. 2008; 98:186–97. https://doi.org/10.1016/j.pbiomolbio.2008.10.010.
3. Workman AJ, Kane KA, Rankin AC. The contribution of ionic currents to changes in refractoriness of human atrial myocytes associated with chronic atrial fibrillation. Cardiovasc Res. 2001; 52:226–35.
4. Gima K, Rudy Y. Ionic current basis of electrocardiographic waveforms: a model study. Circ Res. 2002; 90:889–96.
5. Quan W, Rudy Y. Unidirectional block and reentry of cardiac excitation: a model study. Circ Res. 1990; 66:367–82.
6. El Harchi A, McPate MJ, Zhang Y, Zhang H, Hancox JC. Action potential clamp and chloroquine sensitivity of mutant Kir2.1 channels responsible for variant 3 short QT syndrome. J Mol Cell Cardiol. 2009; 47:743–7. https://doi.org/10.1016/j.yjmcc.2009.02.027.
7. Adeniran I, El Harchi A, Hancox JC, Zhang H. Proarrhythmia in KCNJ2-linked short QT syndrome: insights from modelling. Cardiovasc Res. 2012; 94:66–76. https://doi.org/10.1093/cvr/cvs082.
8. ten Tusscher KH, Panfilov AV. Alternans and spiral breakup in a human ventricular tissue model. Am J Physiol Heart Circ Physiol. 2006; 291:H1088–100. https://doi.org/10.1152/ajpheart.00109.2006.
9. O'Hara T, Virag L, Varro A, Rudy Y. Simulation of the undiseased human cardiac ventricular action potential: model formulation and experimental validation. PLoS Comput Biol. 2011; 7:e1002061. https://doi.org/10.1371/journal.pcbi.1002061.

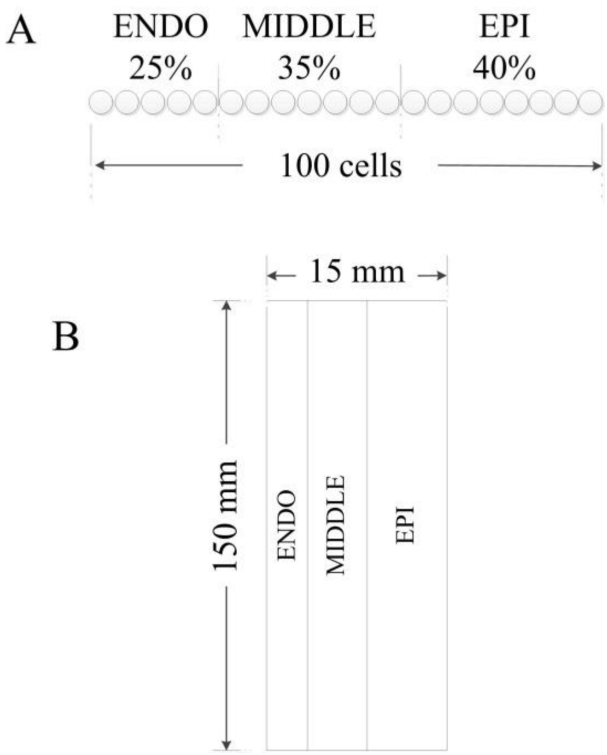

Supplementary Figure 1: Schematic representation of the 1D strand and 2D idealized models. (**A**) 1D transmural ventricular strand model. (**B**) Schematic representation of the 2D idealized model.

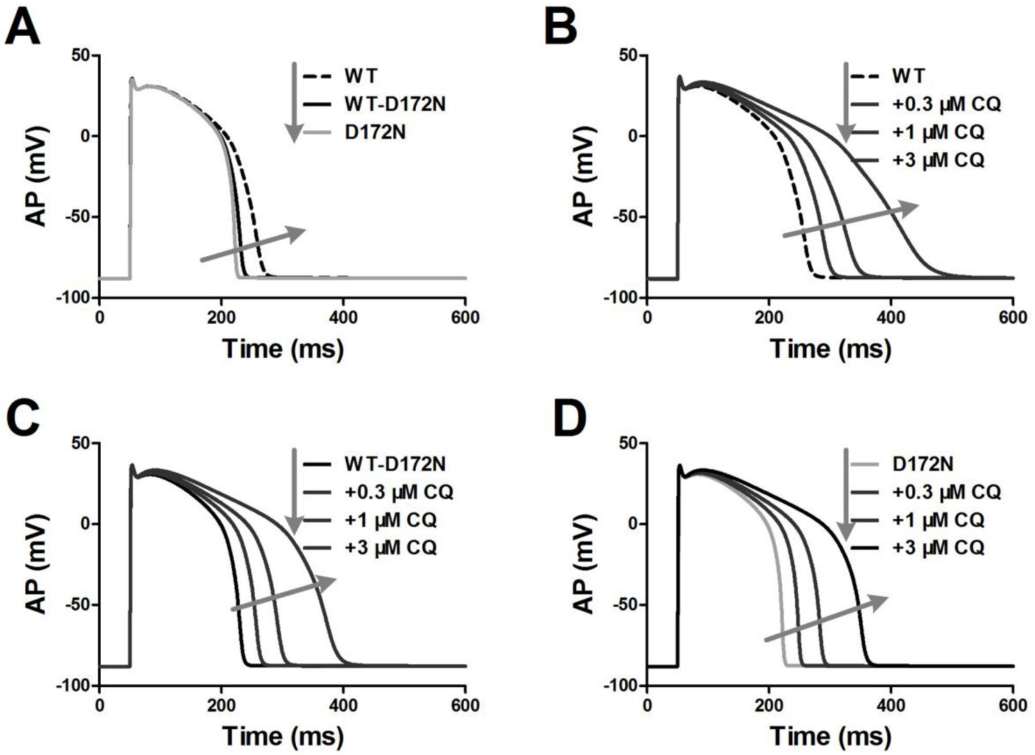

Supplementary Figure 2: Transmembrane voltages of an EPI myocyte from the ORd model for WT, WT-D172N and D172N simulations at the indicated CQ concentrations (0.3, 1 and 3 μM). (**A**) AP waveforms for WT, WT-D172N and D172N conditions. (**B**) AP waveforms for WT simulation at the indicated CQ concentrations. (**C**) AP waveforms for WT-D172N simulation at the indicated CQ concentrations. (**D**) AP waveforms for D172N simulation at the indicated CQ concentrations.

Supplementary Video 1: WT re-entry in an idealized 2D geometry. Initiation and conduction of re-entrant excitation wave in an idealized 2D model of the human transmural ventricle in the WT condition. A planar wave generated by S1 stimulus at the ENDO side propagates through MIDDLE and then towards the EPI end. An S2 stimulus is applied to the MIDDLE-EPI junction during the vulnerable window at 340 ms, which develops into a re-entrant excitation wave. The re-entrant excitation wave self-terminated within 2000 ms. See Supplementary_Video_1

Supplementary Video 2: WT-D172N re-entry in an idealized 2D geometry. Initiation and conduction of re-entrant excitation wave in an idealized 2D model of the human transmural ventricle in the WT-D172N condition. A planar wave generated by S1 stimulus at the ENDO side propagates through MIDDLE and then towards the EPI end. An S2 stimulus is applied to the MIDDLE-EPI junction during the vulnerable window at 314 ms, which develops into a re-entrant excitation wave. The re-entrant excitation wave persisted in the WT-D172N condition. See Supplementary_Video_2

Supplementary Video 3: WT-D172N + 3 μM CQ re-entry in an idealized 2D geometry. Initiation and conduction of re-entrant excitation wave in an idealized 2D model of the human transmural ventricle in the WT condition. A planar wave generated by S1 stimulus at the ENDO side propagates through MIDDLE and then towards the EPI end. An S2 stimulus is applied to the MIDDLE-EPI junction during the vulnerable window at 340 ms, which develops into a re-entrant excitation wave. The re-entrant excitation wave terminated within 2000 ms. See Supplementary_Video_3

Supplementary Video 4: WT re-entry in a realistic 2D geometry. Re-entrant excitation wave generated by the application of a premature S2 stimulus into the refractory and partially recovered region of a wave after a delay of 340 ms from the initial wave stimulus in the WT condition. The induced re-entrant waves transition from transmural re-entry with tip rotating within the wall to anatomical re-entry with tip rotating around the boundary. The re-entry self-terminated within 2000 ms. See Supplementary_Video_4

Supplementary Video 5: WT-D172N re-entry in a realistic 2D geometry. Re-entrant excitation wave generated by the application of a premature S2 stimulus into the refractory and partially recovered region of a wave after a delay of 310 ms from the initial wave stimulus in the WT-D172N condition. The induced re-entrant excitation waves transition from transmural re-entry with tip rotating within the wall to anatomical re-entry with tip rotating around the boundary. The re-entry persisted in the WT-D172N condition. See Supplementary_Video_5

Supplementary Video 6: WT-D172N + 3 μM CQ re-entry in a realistic 2D geometry. Re-entrant excitation wave generated by the application of a premature S2 stimulus into the refractory and partially recovered region of a wave after a delay of 340 ms from the initial wave stimulus in the WT-D172N + 3 μM CQ condition. The induced re-entrant excitation waves transition from transmural re-entry with tip rotating within the wall to anatomical re-entry with tip rotating around the boundary. The re-entry terminated in the WT-D172N + 3 μM CQ condition. See Supplementary_Video_6

Supplementary Video 7: WT re-entry in a realistic 3D geometry. Re-entrant scroll wave was initiated by using the cut-wavefront protocol after a delay of 210 ms for WT condition from the initial wave stimulus. The scroll wave self-terminated within 1000 ms. See Supplementary_Video_7

Supplementary Video 8: WT-D172N re-entry in a realistic 3D geometry. Re-entrant scroll wave was initiated by using the cut-wavefront protocol after a delay of 190 ms for the WT-D172N condition from the initial wave stimulus. The scroll wave persisted under the WT-D172N condition. See Supplementary_Video_8

Supplementary Video 9: WT-D172N + 3 μM CQ re-entry in a realistic 3D geometry. Re-entrant scroll wave was initiated by using the cut-wavefront protocol after a delay of 210 ms for WT-D172N + 3 μM CQ condition from the initial wave stimulus. The scroll wave terminated within 1000 ms. See Supplementary_Video_9
